# Exploring the influencing factors of the pathologic complete response in estrogen receptor-positive, HER2-negative breast cancer after neoadjuvant chemotherapy: a retrospective study

**DOI:** 10.1186/s12957-022-02492-7

**Published:** 2022-01-29

**Authors:** Lingfeng Tang, Xiujie Shu, Gang Tu

**Affiliations:** The First Affiliated Hospital of Chongqing Medical University, No. 1 Friendship Road, Yuzhong District, Chongqing, 400000 China

**Keywords:** Breast cancer, Neoadjuvant chemotherapy, Pathologic complete response, Nomogram

## Abstract

**Background:**

Pathological complete response (pCR) is the goal of neoadjuvant chemotherapy (NACT). We aimed to develop a nomogram to predict the probability of achieving pCR in estrogen receptor-positive (ER+), HER2-negative (HER2−) breast cancer patients.

**Methods:**

A total of 273 ER+, HER2− breast cancer patients who received 4 cycles of thrice-weekly standard NACT in the First Affiliated Hospital of Chongqing Medical University were retrospectively enrolled. Univariate and multivariate logistic regression analyses were used to screen the predictive factors to develop the nomograms. The discrimination and calibration abilities were assessed by the C-index, receiver operating characteristic curve (AUC), and calibration plot.

**Results:**

There were 28 patients (10.3%) with overall pCR, 38 patients (13.9%) with breast pCR after NACT. ER expression, PgR expression, the neutrophil-to-lymphocyte ratio (NLR) and the Ki-67 index were independent predictive factors for achieving overall pCR. These indicators had good discrimination and calibration ability (AUC 0.843). The nomogram for breast pCR was established based on ER expression, PgR expression, the NLR, and the Ki-67 index and showed great discriminatory ability, with an AUC of 0.810. The calibration curve showed that the predictive ability of the nomogram was a good fit to actual observations.

**Conclusion:**

The nomograms exhibited a sufficient discriminatory ability for predicting pCR after NACT in ER+, HER2− breast cancer patients. Utilizing these nomograms will enable us to identify patients at high probability for pCR after NACT and provide a reference for preoperative adjuvant therapy.

**Supplementary Information:**

The online version contains supplementary material available at 10.1186/s12957-022-02492-7.

## Introduction

Breast cancer has become the most common cancer among women in the world. Compared with developed countries, more patients in China are diagnosed with advanced breast cancer (ABC) [1]. Primary locally advanced breast cancer (LABC) traditionally refers to inoperable nonmetastatic locally advanced breast cancer, including T3/T4 tumors (diameter > 5 cm or invasion of the skin and chest wall), N2 axillary nodes, ipsilateral supraclavicular lymph node metastasis, and IBC [2]. Neoadjuvant chemotherapy (NACT), which is utilized before surgery and radiotherapy, is mainly used for the management of patients with LABC. By killing some proliferative and active cancer cells, NACT can effectively reduce the clinical stage of breast cancer, making inoperable breast cancer operable breast cancer or increasing the chances of breast conservation [3]. The benefits of NACT also include the in vivo assessment of the response to chemotherapy. Many studies have demonstrated that patients who achieve a pathological complete response (pCR) after NACT seem to have improved long-term outcomes [3–5].

Nonetheless, not all patients can benefit from NACT. Based on the estrogen receptor (ER), progesterone receptor (PgR), and human epidermal growth factor receptor 2 (HER2), four main major breast cancer subtypes have been identified, including luminal A and luminal B, basal-like and HER2-enriched [6]. Estrogen receptor-positive (ER+) breast cancer, accounting for approximately 50 ~ 60% of breast cancer cases, with a pCR rate of nearly 10%, is relatively insensitive to chemotherapy treatment compared to other subtypes [7]. A study on NACT for stage I to III breast cancer patients at the MD Anderson Cancer Center reported that patients with basal-like breast cancer had significantly higher pCR rates than non-TNBC patients (22% vs. 11%; *P* = 0.034) [8]. Patricia et al. reviewed 12 international NACT trials, including 13856 patients, and found a significantly poor response to chemotherapy in those with ER+ breast cancer [9]. The pCR rate of ER+ breast cancer after NACT was less than half of that of hormone receptor-negative (HR−) breast cancer. Therefore, blindly receiving NACT in patients with ER+ breast cancer may lead to worsening of the disease, and these patients may suffer from toxicities and adverse reactions associated with chemotherapy. There is a need for reliable predictors of chemosensitivity in ER+ breast cancer patients that can enable the screening of those who can benefit from NACT.

Studies have demonstrated the correlation between clinical pathological characteristics and chemotherapy efficacy [10]. For instance, the level of ER expression and the proliferation index of Ki-67 are closely linked to chemosensitivity [11, 12]. Some clinical features are also related to the efficacy of chemotherapy, including tumour size, BMI, and axillary lymph node metastasis [13]. Furthermore, the tumour microenvironment is relevant to the development and metastasis of breast cancer, and in this environment, the immune and inflammatory responses play an important role [14]. Some evidence has suggested that the pretreatment neutrophil-to-lymphocyte ratio (NLR) and the platelet-to-lymphocyte ratio (PLR) reflect the inflammatory response and efficacy of chemotherapy [15–17].

The purpose of this study was to screen the influencing factors of NACT and introduce them into the prediction model for predicting the probability of achieving pCR in patients with ER+, HER2− breast cancer. Such a nomogram would be useful in evaluating sensitivity to chemotherapy, which can provide a reference for clinical treatment.

## Methods

### Population

We accessed the database and screened patients admitted between 6 May 2020 and 31 May 2020. The database was reviewed to identify all patients diagnosed from the First Affiliated Hospital of Chongqing Medical University between 1 January 2012 and 31 December 2019. We used the following inclusion criteria: (I) female; (II) no antitumor treatment performed before NACT; (III) ER+ invasive ductal breast cancer; and (IV) complete data. The exclusion criteria were as follows: (I) inflammatory breast cancer; (II) infiltrating lobular carcinoma; (III) other primary tumours; (IV) bilateral breast cancer; and (V) HER2-positive (HER2+) breast cancer. Informed consent was obtained from each patient prior to treatment. All histological specimens were paraffin-embedded and evaluated by two skilled pathologists. This study was approved by the Ethics Committee of the First Affiliated Hospital of Chongqing Medical University (No. 2020-202). This article does not refer to the privacy of patients, so informed consent was exempted. All data were fully anonymized before we accessed them. The authors were not provided with information that could identify individual participants during or after data collection.

### Clinicopathologic analysis

Data on the medical history, concurrent diseases, age, menopausal status, body mass index (BMI), tumor location, histological grade, tumor size, blood supply of tumor, lymph node (LN) status, NLR, PLR, fibrinogen (FBG) in peripheral blood, hormone receptor (HR) status, p53 status, Ki-67 index, and NACT regimens were estimated before NACT. The cut-off values of NLR, PLR and FBG were evaluated by the largest Youden index [18]. Clinical assessments of the breast, including preoperative LN status, tumor size, and blood supply around the tumour, depended on MRI or breast ultrasonography. RECIST criteria were used for the clinical response evaluation [19]. The ER, PgR, p53, and Ki-67 statuses were evaluated by immunohistochemistry (IHC) of the pretreatment core biopsy specimens. Cancers with 1–100% of cells positive for ER/PgR expression were considered ER-positive/PgR-positive. The Ki-67 index was defined as the percentage of the total number of tumour cells (at least 1000) with nuclear staining over 10 high powered fields (× 40). Then, overall pCR was defined as no residual invasive cancer in the breast or evidence of disease in the axillary lymph nodes (ypT0ypN0) after NACT. And breast pCR was defined as no residual invasive cancer in the breast after NACT.

### Treatment

The criteria for receiving neoadjuvant chemotherapy in HR (+) breast cancer patients were as follows: the local stage of the disease was relatively late, such as patients with axillary lymph node metastasis or large mass or invasion of skin and chest wall, as well as patients who had a strong desire to do breast-conserving surgery but did not meet the indication of breast-conserving surgery when diagnosed.

NACT was given according to the local protocol and national guidelines. The treatments were predominantly anthracycline and taxane, and the TEC (docetaxel 75 mg/m^2^, epirubicin 75 mg/m^2^, and cyclophosphamide 500 mg/m^2^) NACT regimens were administered every 3 weeks. After diagnosis, all patients started the first cycle of NACT in a week and received four cycles of NACT regimens.

### Statistical methods

Statistical analysis was performed by R software (Version 4.0.2) and SPSS (Version 25.0). The best cut-off values were determined by the largest Youden index. Categorical variables were compared using the chi-squared test or Fisher’s exact test. Then, univariable and multivariable logistic regression analyses were used to screen out the independent predictors. To quantify the discrimination performance of the nomogram, Harrell’s C-index was measured. The intolerant abilities of the model were assessed by measuring the area under the receiver operating characteristic (ROC) curve. Calibration curves were plotted to assess the calibration of the nomogram [20]. In this case, the calibration is the agreement between the frequencies of the observed outcomes and the probabilities predicted by the model. *P* < 0.05 was defined as statistically significance.

## Results

### The relationship between the clinicopathological characteristics and different outcomes

A total of 273 patients with ER+, HER2− breast cancer who received NACT were identified and evaluated (mean age 49.77 ± 9.97 years [range 24–79 years]). The optimal cut-off values were 45 for age, 117.88 for PLR, 1.73 for NLR and 2.46 for fibrinogen level. There were 200 patients (73.3%) who achieved cPR, 28 patients (10.3%) with overall pCR, and 38 patients (13.9%) with breast pCR after NACT.

We compared the clinicopathological characteristics of patients with different outcomes (overall pCR vs. overall non-pCR; breast pCR vs. breast non-pCR), and the results are displayed in Table 1. Overall pCR and breast pCR were significantly associated with histological grade, ER, PgR, and Ki-67 (*P* < 0.05).

**Table 1:** Baseline clinicopathological characteristics of patients with ER+, Her2− breast cancer (*n* = 273)

Characteristic	n(%)	Overall			Breast		
	Total (*n* = 273)	pCR (*n* = 28)	Non-pCR (*n* = 245)	*P* value^a^	pCR (*n* = 38)	Non-pCR (*n* = 235)	*P* value
**Clinical variable**							
Age (years)				0.868			0.907
< 45	84 (30.8)	9 (32.1)	75 (30.6)		12 (31.6)	72 (30.6)	
≥ 45	189 (69.2)	19 (67.9)	170 (69.4)		26 (68.4)	163 (69.4)	
BMI (kg/m^2^)				0.975			0.388
< 24	147 (53.8)	15 (53.6)	132 (53.9)		18 (47.4)	129 (54.9)	
≥ 24	126 (46.2)	13 (46.4)	113 (46.1)		20 (52.6)	106 (45.1)	
Tumor location				0.822			0.935
Left	131 (48.0)	14 (50.0)	117 (47.8)		18 (47.4)	113 (48.1)	
Right	142 (52.0)	14 (50.0)	128 (52.2)		20 (52.6)	122 (51.9)	
Menopausal status				0.116			0.685
Postmenopausal	116 (42.5)	8 (28.6)	108 (44.1)		15 (39.5)	101 (43.0)	
Premenopausal	157 (57.5)	20 (71.4)	137 (55.9)		23 (60.5)	134 (57.0)	
PLR				0.242			0.069
< 117.88	116 (42.5)	9 (32.1)	107 (43.7)		11 (28.9)	105 (44.7)	
≥ 117.88	157 (57.5)	19 (67.9)	138 (56.3)		27 (71.1)	130 (55.3)	
NLR				0.067			0.283
< 2.46	94 (34.4)	14 (50.0)	80 (32.7)		16 (42.1)	78 (33.2)	
≥ 2.46	179 (65.6)	14 (50.0)	165 (67.3)		22 (57.9)	157 (66.8)	
FBG				0.392			0.225
< 1.73	39 (14.3)	2 (7.1)	37 (15.1)		35 (92.1)	199 (84.7)	
≥ 1.73	234 (85.7)	26 (92.9)	208 (84.9)		3 (7.9)	36 (15.3)	
Tumor size (cm)							0.988
< 2	35 (12.8)	4 (14.3)	31 (12.7)	0.954	5 (13.2)	30 (12.8)	
2 ~ 5	195 (71.4)	20 (71.4)	175 (71.4)		27 (71.1)	168 (71.5)	
> 5	43 (15.8)	4 (14.3)	39 (15.9)		6 (15.8)	37 (15.7)	
Lymph node status				0.854			0.406
cN0	132 (48.4)	14 (50.0)	118 (48.2)		16 (42.1)	116 (49.4)	
cNX	141 (51.6)	14 (50.0)	127 (51.8)		22 (57.9)	119 (50.6)	
Blood supply				0.145			0.943
Poor	102 (37.4)	14 (50.0)	88 (35.9)		14 (36.8)	84 (37.4)	
Abundant	171 (62.6)	14 (50.0)	157 (64.1)		24 (63.2)	147 (62.6)	
**Pathological variable**							
Histological grade				**0.038**			**0.009**
I	20 (7.3)	1 (3.6)	19 (7.8)		2 (5.3)	18 (7.7)	
II	229 (83.9)	21 (75.0)	208 (84.9)		27 (71.1)	202 (84.0)	
III	24 (8.8)	6 (21.4)	18 (7.3)		9 (23.6)	15 (6.3)	
ER (%)				**0.002**			**0.005**
[1, 10]	16 (5.9)	5 (17.9)	11 (4.5)		6 (15.8)	10 (4.3)	
[10, 30]	17 (6.2)	2 (7.1)	15 (6.1)		4 (10.5)	13 (5.5)	
[30, 50]	30 (11.0)	7 (25.0)	23 (9.4)		8 (21.1)	22 (9.4)	
[50, 70]	60 (22.0)	6 (21.4)	54 (22.0)		8 (21.1)	52 (22.1)	
> 70	150 (54.9)	8 (28.6)	142 (58.0)		12 (31.5)	138 (58.7)	
PgR(%)				**0.003**			**<0.001**
Negative	62 (22.7)	8 (28.6)	54 (22.0)		12 (31.6)	50 (21.3)	
[1, 10]	40 (14.7)	10 (35.7)	30 (12.3)		13 (34.2)	27 (11.5)	
> 10	171 (62.6)	10 (35.7)	161 (65.7)		13 (34.2)	158 (67.2)	
Ki-67(%)				**0.005**			**<0.001**
< 20	101 (37.0)	3 (10.7)	98 (40.0)		5 (13.2)	96 (40.9)	
[20, 40)	110 (40.3)	13 (46.4)	97 (39.6)		15 (39.4)	95 (40.3)	
[40, 60)	43 (15.8)	8 (28.6)	35 (14.3)		13 (34.2)	30 (12.8)	
≥ 60	19 (7.0)	4 (14.3)	15 (6.1)		5 (13.2)	14 (6.0)	
P53 status				0.424			0.163
Negative	76 (27.8)	6 (21.4)	70 (28.6)		7 (18.4)	69 (29.4)	
Positive	197 (72.2)	22 (78.6)	175 (71.4)		31 (81.6)	166 (70.6)	

*pCR* pathologic complete response, *BMI* body mass index, *PLR* platelet to lymphocyte ratio, *NLR* neutrophil to lymphocyte ratio, *FBG* fibrinogen, *ER* estrogen receptor, *PgR* progesterone receptor, *HER2* human epidermal growth factor receptor2, *ER+* estrogen receptor-positive

^a^*P* values were determined by chi-square tests. Bold values indicate statistical significance (*P* < 0.05)

### Prediction models for overall pCR in ER+, HER2− breast cancer

Based on univariate analysis, there were significant differences in ER (*P* = 0.006), PgR (*P* = 0.003), and Ki-67 (*P* = 0.022) for overall pCR. Then, we included the factors (*P* < 0.1) in the multivariate analysis. We found that NLR (*P* = 0.020), ER (*P* = 0.011), PgR (*P* = 0.002) and Ki-67 (*P* = 0.011) were independent predictors of overall pCR (Table 2).

**Table 2:** Univariate and multivariate analysis of overall pCR in patients with ER+, Her2− breast cancer (*n* = 273)

Characteristics	Univariate analysis OR (95% CI)	*P* value	Multivariate analysis OR (95% CI)	*P* value
Age, years (≥ 45 vs < 45)	0.931 (0.403–2.154)	0.868		–
BMI, kg/m^2^ (≥ 24 vs < 24)	1.012 (0.462–2.217)	0.975		–
Tumor location (right vs left)	0.914 (0.418–1.998)	0.822		–
Menopausal status (premenopausal vs postmenopausal)	1.971 (0.836–4.647)	0.121		–
PLR (≥ 117.88 vs < 117.88)	1.637 (0.712–3.763)	0.246		–
NLR (≥ 2.46 vs < 2.46)	0.485 (0.221–1.066)	0.072	0.335 (0.133–0.843)	**0.020**
FBG (≥ 1.73 vs < 1.73)	2.312 (0.526–10.160)	0.267		–
Tumor size, cm		0.954		–
< 2	1 (reference)			
2 ~ 5	0.886 (0.283–2.768)			
> 5	0.795 (0.184–3.436)			
Lymph node status (cNX vs cN0)	0.929 (0.425–2.031)	0.854		–
Blood supply (abundant vs poor)	0.561 (0.256–1.229)	0.149		–
Histological grade		0.052		0.821
I	1 (reference)		1 (reference)	
II	1.918 (0.244–15.056)		1.542 (0.162–14.664)	
III	6.333 (0.693–57.905)		2.070 (0.174–24.651)	
ER (%)		**0.006**		**0.011**
[1, 10]	1 (reference)		1 (reference)	
[10, 30]	0.293 (0.048–1.801)		0.117 (0.023–1.377)	
[30, 50]	0.670 (0.173–2.593)		0.673 (0.138–3.270)	
[50, 70]	0.244 (0.063–0.945)		0.289 (0.056–1.509)	
> 70	0.124 (0.035–0.443)		0.085 (0.017–0.433)	
PgR (%)		**0.003**		**0.006**
Negative	1 (reference)		1 (reference)	
[1, 10]	2.250 (0.802–6.310)		4.790 (1.316–17.431)	
> 10	0.419 (0.157–1.116)		0.932 (0.272–3.191)	
Ki-67(%)		**0.022**		**0.011**
< 20	1 (reference)		1 (reference)	
[20, 40)	4.378 (1.210–15.847)		6.964 (1.682–28.839)	
[40, 60)	7.467 (1.875–29.735)		11.316 (2.475–51.729)	
≥ 60	8.711 (1.772–42.825)		14.444 (2.168–96.222)	
P53 status (positive vs negative)	1.467 (0.570–3.771)	0.427		–

**Fig. 1:**
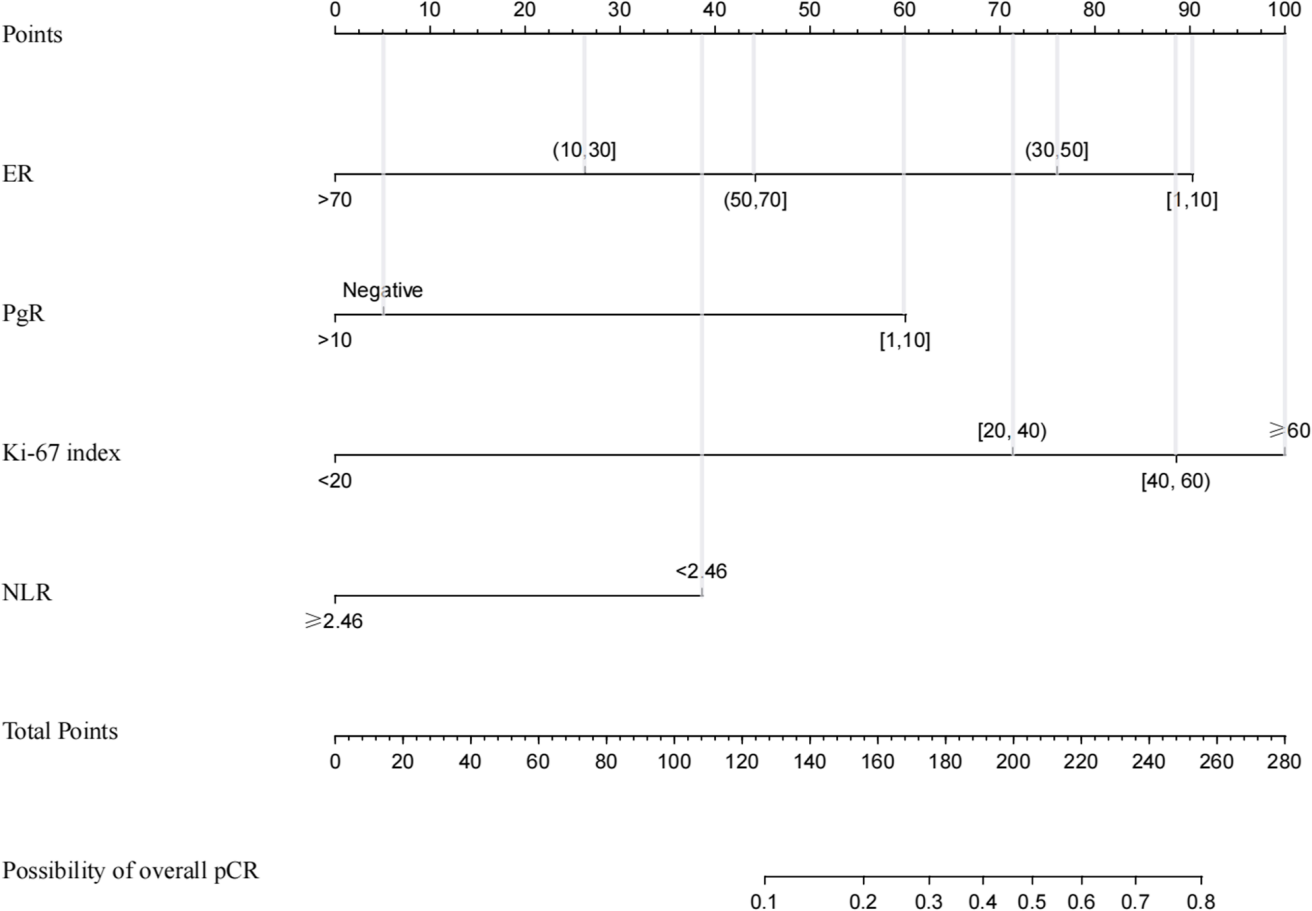
Nomogram for predicting overall pCR in ER+, HER2− breast cancer patients after NACT. A line is drawn straight up to the point axis that corresponds with each patient variable to obtain the points. The sum of these points is located on the total score points axis. A line is drawn downwards to the risk axis to determine the possibility of achieving overall pCR

The prediction model incorporating the above independent predictors was developed by R software (Fig. 1). The nomogram shows the score of each predictor on the upper scale. Therefore, the probability of overall pCR is determined by the total points of all predictors. Inputting the necessary clinicopathological data concisely estimated the probability of overall pCR after NACT.

**Fig. 2:**
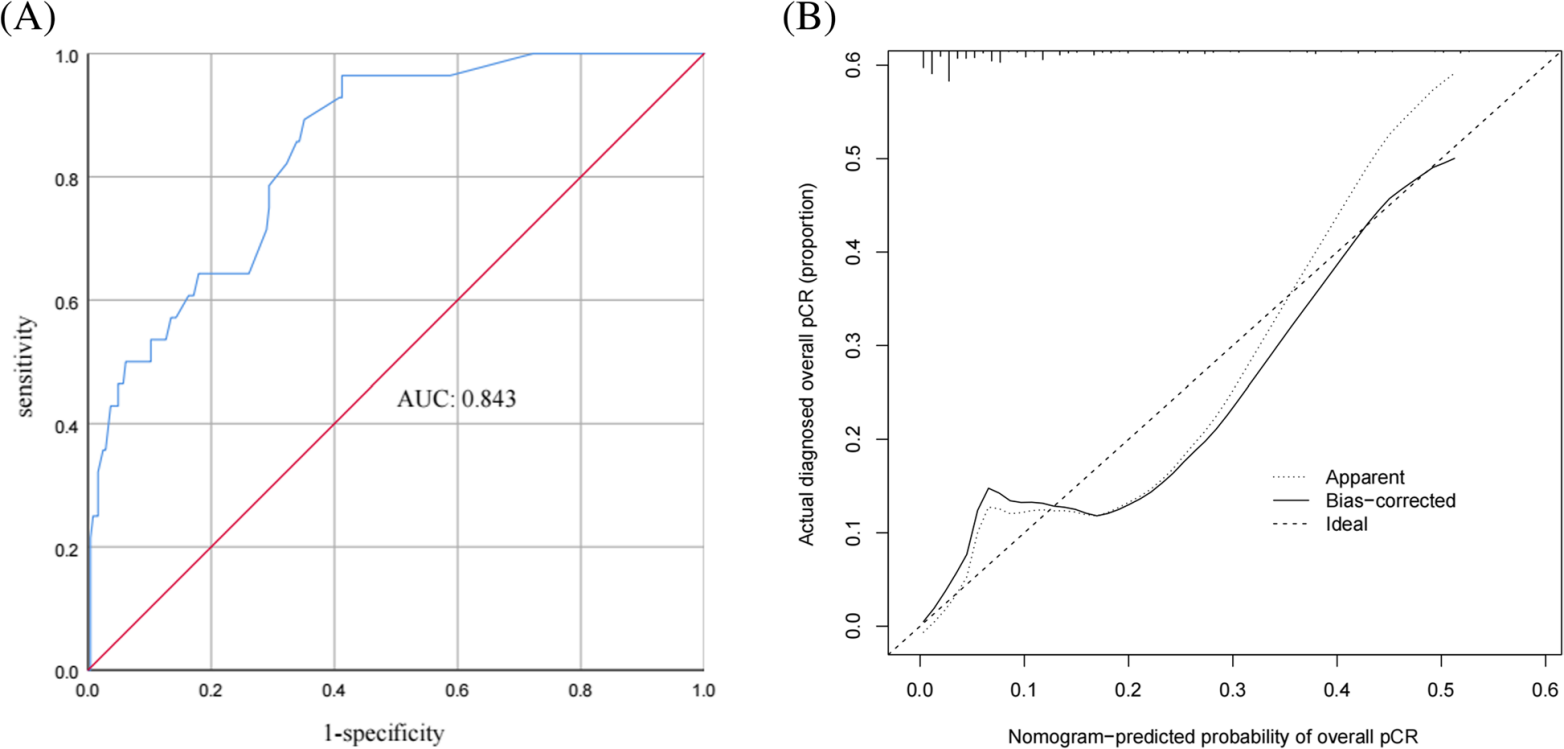
**A** Receiver operating characteristic curve (AUC 0.843). **B** The calibration plot depicts the calibration of the model in terms of the agreement between the predicted possibility of overall pCR and the observed outcomes of overall pCR

According to this model, the ROC curve was drawn (Fig. 2A), and the area under the curve (AUC) was 0.843 (95% CI 0.775–0.911). The C-index of the prediction models was 0.843, which demonstrates good discriminative ability. The calibration plot revealed good agreement between the predictions and actual observations (Fig. 2B). These results show that this nomogram has good efficacy in predicting the probability of overall pCR in ER+, HER2− breast cancer.

### Prediction models for breast pCR in ER+, Her2− breast cancer

**Fig. 3:**
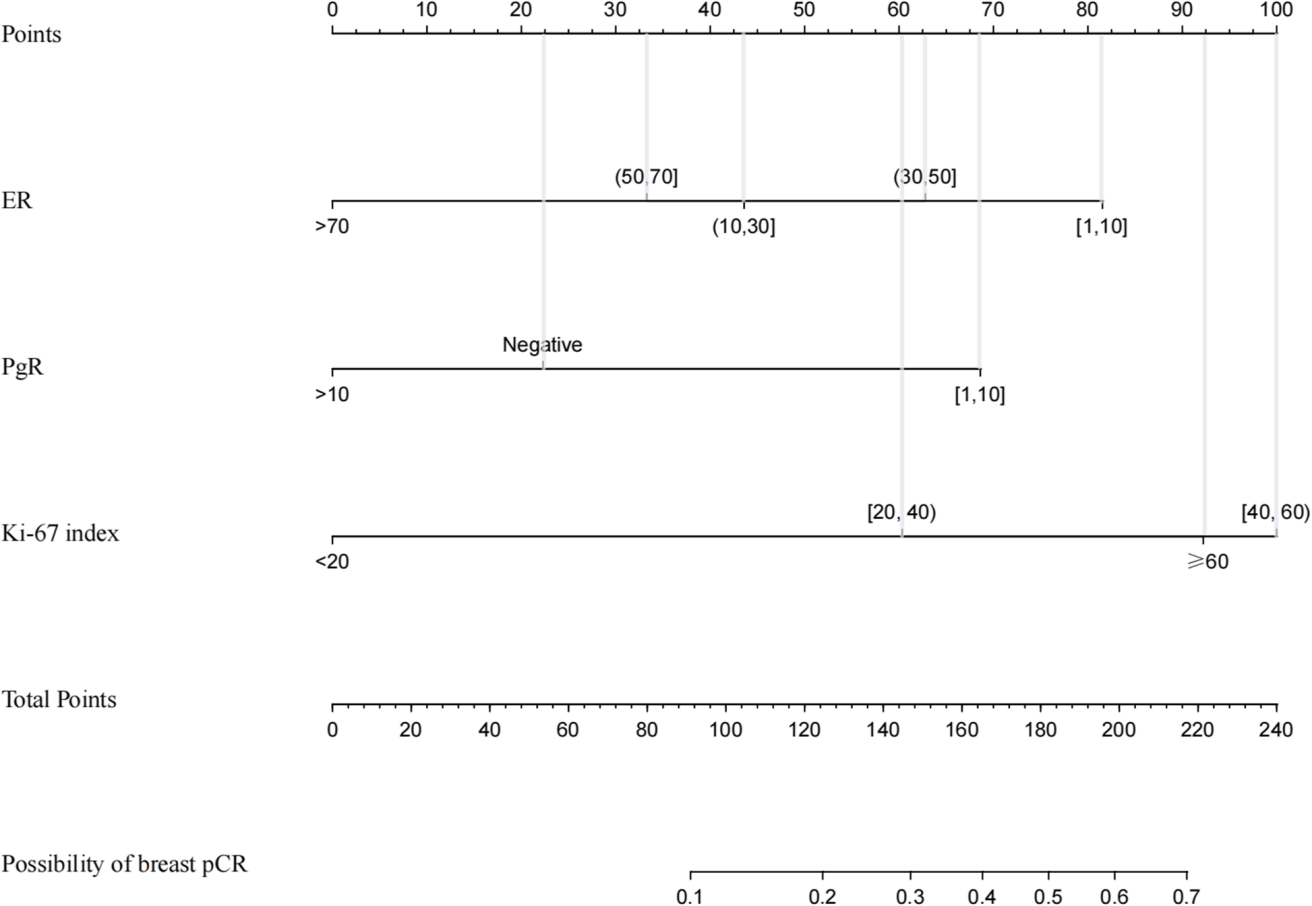
Nomogram for predicting breast pCR in ER+, HER2− breast cancer patients after NACT

The results of the univariate and multivariate logistic regression analyses are displayed in Table 3. ER, PgR and the Ki-67 index were indicated as independent predictors for breast pCR in ER+, HER2− breast cancer patients. Patients with lower ER expression, a higher Ki-67 index and a PgR expression rate in the range of 0–10% were more likely to achieve breast pCR. Then, we established a nomogram to predict breast pCR after NACT (Fig. 3).

**Table 3:** Univariate and multivariate analysis of breast pCR in patients with ER+, Her2− breast cancer (*n* = 273)

Characteristics	Univariate analysis OR (95% CI)	*P* value	Multivariate analysis OR (95% CI)	*P* value
Age, years (≥ 45 vs < 45)	0.957 (0.457–2.002)	0.907		–
BMI, kg/m^2^ (≥ 24 vs < 24)	1.352 (0.680–2.687)	0.389		–
Tumor location (right vs left)	1.029 (0.518–2.044)	0.935		–
Menopausal status (premenopausal vs postmenopausal)	1.156 (0.574–2.327)	0.685		–
PLR (≥ 117.88 vs < 117.88)	1.983 (0.940–4.183)	0.072	2.008 (0.855–4.719)	0.110
NLR (≥ 2.46 vs < 2.46)	0.683 (0.340–1.374)	0.285		–
FBG (≥ 1.73 vs < 1.73)	2.111 (0.616–7.231)	0.234		–
Tumor size, cm		0.998		–
< 2	1 (reference)			
2 ~ 5	0.964 (0.344–2.702)			
> 5	0.973 (0.270–3.502)			
Lymph node status (cNX vs cN0)	1.340 (0.670–2.680)	0.407		–
Blood supply (abundant vs poor)	1.026 (0.504–2.088)	0.943		–
Histological grade		**0.005**		0.359
I	1 (reference)		1 (reference)	
II	1.203 (0.264–5.473)		0.969 (0.168–5.598)	
III	5.400 (1.008–28.928)		2.152 (0.291–15.904)	
ER (%)		**0.004**		**0.020**
[1, 10]	1 (reference)		1 (reference)	
[10, 30]	0.513 (0.113–2.322)		0.400 (0.074–2.169)	
[30, 50]	0.606 (0.166–2.215)		0.636 (0.151–2.670)	
[50, 70]	0.256 (0.073–0.901)		0.312 (0.071–1.367)	
> 70	0.145 (0.045–0.468)		0.140 (0.036–0.545)	
PgR (%)		**< 0.001**		**0.003**
Negative	1 (reference)		1 (reference)	
[1, 10]	2.006 (0.805–5.002)		4.790 (1.316–17.431)	
> 10	0.343 (0.147–0.799)		0.932 (0.272–3.191)	
Ki-67 (%)		**0.001**		**0.001**
< 20	1 (reference)		1 (reference)	
[20, 40]	3.032 (1.060–8.673)		4.290 (1.363–13.498)	
[40, 60]	8.320 (2.742–25.243)		11.174 (3.306–37.771)	
≥ 60	6.857 (1.759–26.730)		9.262 (1.959–43.781)	
P53 status (positive vs negative)	1.841 (0.774–4.380)	0.168		–

**Fig. 4:**
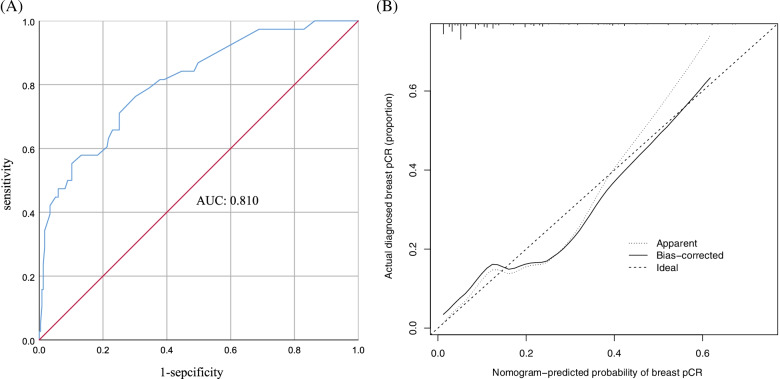
**A** Receiver operating characteristic curve (AUC 0.810). **B** Calibration plot of the nomogram

The AUC was 0.810 (95% CI 0.734–0.885), and the C-index of this model was 0.808 (Fig. 4A). Good agreement between the predictions and actual observations was also shown in the calibration plot (Fig. 4B). These results demonstrate that this nomogram has good discriminative ability and efficacy in predicting the probability of breast pCR in ER+, HER2− breast cancer.

## Discussion

Currently, the widespread use of NACT in the treatment of breast cancer leads to an increase in the breast conservation rate and a reduction in the recurrence rate. Furthermore, robust evidence demonstrates that achieving pCR after NACT indicates a good prognosis [21, 22]. ER+ breast cancer is relatively insensitive to chemotherapeutics, which causes many patients to suffer side effects of chemotherapy without the expected efficacy. For those in whom clinical down staging is not the goal, it is imperative to determine who is more likely to achieve pCR after NACT in ER+ breast cancer patients. At present, targeted drugs in preoperative treatment have greatly improved the pCR rates and prognosis of HER2+ patients [23]. For the accuracy of our study, we only included ER+, HER2− breast cancer patients.

Currently, the expression of ER, PgR, HER2 and Ki-67 has become a routine pathological indicator. By immunohistochemistry, breast cancer can be quickly classified into five categories, and the degree of malignancy can be preliminarily judged. In our study, on the basis of univariate and multivariate logistic analyses, ER expression, PgR expression, and the Ki-67 index were screened as independent prognostic predictors of achieving breast pCR or overall pCR in ER+, HER2− breast cancer patients. We found a higher pCR rate in patients with low ER expression and low PgR expression. In addition, we subdivided the expression of ER into 5 levels and were able to judge the chemosensitivity of patients accurately in our study. The patients with ≤ 10% ER expression had extremely high possibility (31.3%) of achieving overall pCR, in comparison, the pCR rate of the other patients was only 8.9%. With the similar characteristics of basal-like breast cancer, the patients with ≤ 10% ER expression may obtain excellent benefit from NACT. For the patients with higher ER expression, we need to consider more factors. Moreover, Petruolo et al. conducted a retrospective analysis of 402 ER+, HER2− breast cancer patients that assessed the rates of pCR and found a significantly lower rate of pCR in PgR-positive patients, which is also consistent with our results [24]. Our results illustrated that the patients with low (≤ 10%) or negative expression of PgR both had higher possibility of achieving pCR than whom with high expression of PgR. Other investigators have found that the expression level of Ki-67 reflects the ability of tumour cell proliferation, which is closely related to the sensitivity to chemotherapy [25]. A study of 122 breast cancer patients showed that Ki-67 was the only independent influencing factor of achieving pCR in ER+ breast cancer patients after NACT (OR = 6.24, 95% CI 1.40 ~ 27.7, *P* = 0.016) [26]. Among the ER+, HER2− breast cancer patients in their study, 14.0% achieved pCR. Shuai analyzed 268 ER+ patients, and the overall pCR rate was 4.1%. They obtained an approximately similar conclusion that higher Ki-67 expression and more NACT cycles were associated with higher pCR rates [27].

In our study, we developed and validated two easy-to-use nomograms to predict overall pCR and breast pCR after thrice weekly standard NACT in ER+, HER2− breast cancer patients. The AUC and C-index were greater than 0.8, which suggests that the models have predictive power. Through this nomogram, we can quickly understand the sensitivity of a patient to chemotherapy and the likelihood of achieving overall pCR or breast pCR. The clinicopathological factors that influence the likelihood of achieving breast pCR, and overall pCR in this study were available before NACT. Using these models, we can understand the probability of pCR after NACT when the patient is first hospitalized.

There are many factors that may influence the efficacy of NACT. In our study, age and menstrual status were not independent influencing factors of achieving a pCR after NACT in ER+, HER2− breast cancer patients, which was consistent with other studies [13, 27]. However, a previous study demonstrated that young breast cancer patients are more likely to achieve pCR after NACT [28]. Additionally, Omranipour et al. found that there is a strong possibility that patients younger than 50 achieve a pCR after NACT [29]. Chemotherapy-induced amenorrhea (CIA), considered to be one of the indicators of good prognosis, which perform an ovarian suppression effect in these younger patients [30]. The estrogen-deprivation induced by NACT may explain the higher pCR rates seen in strongly ER+ patients. Similarly, in our study, a higher pCR rate in the premenopausal patients, unfortunately, we failed to obtain a statistically significant result. Then, similar to the result of a previous study indicated by Alan et al., we concluded BMI was not a predictive biomarker for pCR. But they have suggested that NACT was a poor choice for the ER+, HER2− breast cancer patients with insulin resistance [31].

Recently, the correlations between the PLR, NLR, and FBG and NACT efficacy has remained controversial [15–17, 32]. In our study, we only found that the NLR has predictive value for achieving an overall pCR in ER+, HER2− breast cancer patients. Consistent with our results, Vincenzo et al. also found that a low NLR increases pCR rates by more than two-fold [33]. Nevertheless, the levels of inflammatory cells and mediators are not stable in the human body, which makes these indicators inaccurate in predicting NACT outcomes. Tumor size and node status are important for the formulation of treatment strategies. A large mass (> 5 cm) and axillary lymph node metastasis are both indications of NACT [2]. Consistent with several previous studies, the results of the current study did not find a significant relationship between LN metastasis and NACT outcomes [27, 30]. Hwang et al. showed that patients with stage cN0-1 disease were more likely to achieve pCR than patients with stage cN2-3 disease (OR = 2.93, 95% CI 1.41~6.05, *P* = 0.004) [34]. Since the assessment of LN metastasis was extracted from imaging and physical examination, the lack of biopsy is a major limitation that may cause inaccurate results. Similarly, our study failed to identify tumor size as a predictor of pCR after NACT in ER+, HER2− patients. Nevertheless, achieving pCR is not the only aim of treatment with NACT, other benefits include increasing the eligibility for breast-conserving surgery and decreasing the difficulty of operation [24, 35].

Undoubtedly, the application of chemotherapy in patients with low chemosensitivity will cause medical resource wasting and doctor-patient contradictions. So when we identify a patient who is relatively insensitive to chemotherapy, other therapeutic schemes will be considered. A meta-analysis of 20 studies showed that neoadjuvant endocrine therapy (NAET) combined with NACT for ER+ breast cancer has the same curative effect as NACT and fewer side effects [36]. From a clinical standpoint, NAET represents a feasible and effective treatment option as a substitute for NACT, especially in ER+, HER2− postmenopausal patients [37].

Although the nomogram had a sufficient level of accuracy for predicting pCR in ER+, HER2− breast cancer, these results still need to be confirmed by large external or prospective cohorts. In addition, since we failed to rule out lymph node metastasis by nodal biopsy performed prior to surgery, nodal pCR cannot be assessed in our study. If there is an accurate prediction model for achieving nodal pCR, some patients can avoid axillary lymph node dissection (ALND) after NACT.

## Conclusion

As mentioned above, NACT is generally used in the treatment of breast cancer, but patients with ER+ breast cancer are relatively insensitive to NACT. Understanding the possible outcome of patients after NACT is an important element for determining the treatment plan. Our study developed and validated universally applicable nomograms for achieving overall pCR and breast pCR in ER+, HER2− breast cancer patients. This simple tool will enable oncologists to predict pCR for individual patients after NACT more accurately and identify patients with low chemosensitivity in need of other therapeutic schemes. Nevertheless, more data and validation studies are necessary in the future to further improve this model and provide more accurate guidance for clinical treatment.

## Supplementary Information

**Additional file 1.**

## Data Availability

The datasets generated and analyzed during the current study are available from the corresponding author on reasonable request.
